# Digital storytelling to promote disability-inclusive research in Africa

**DOI:** 10.4102/ajod.v13i0.1495

**Published:** 2024-09-30

**Authors:** Lesley L. Sikapa, Hadiatou Dialo, Veronica N. Ndi, Lanjo S. Neindefoh, Che D. Nkemchap, Lynn Cockburn

**Affiliations:** 1 School of Medicine and Population Health, Faculty of Health, University of Sheffield, Sheffield, United Kingdom; 2 One Family People, Freetown, Sierra Leone; 3 North West Association for Women with Disabilities, (NWAWWD), Bamenda, Cameroon; 4 Department of Performing and Visual Arts, University of Bamenda, Bamenda, Cameroon; 5 Special Needs Entrepreneur Group (SNEG), Bamenda, Cameroon; 6 Department of Occupational Science and Occupational Therapy, Temerty Faculty of Medicine, University of Toronto, Toronto, Canada

**Keywords:** digital storytelling, knowledge translation, knowledge mobilisation, knowledge translation in Africa, disability studies, disability inclusive research, participatory research, arts-based research

## Abstract

**Background:**

Digital stories have been shown to be effective in sharing information. The Partnerships for Inclusive Research and Learning (PIRL) was a 4-year international participatory research project focussed on the digital divide in inclusive research.

**Objectives:**

Members of PIRL share their experience of using digital storytelling to get key messages from the project to a wide range of people.

**Method:**

Members of PIRL were invited to develop digital stories and create project-specific guidelines for digital story development. Seven people participated in workshops given by experts, read literature, watched digital stories and discussed how to create digital stories.

**Results:**

The group created six digital stories, each one addressing a different aspect related to disability-inclusive research, with many having a focus on Africa and the creation of credible African evidence. The importance of assisting community members to think about and support research and evidence creation was one of the goals of the project. The videos provide an avenue to share insights about disability-inclusive development research. Group members stated that being part of the process significantly improved their understanding of translating evidence into formats that are more understandable.

**Conclusion:**

Creating digital stories requires commitment, a significant amount of time, access to digital tools, and financial resources. Working collaboratively on this project was not only meaningful but also encouraged positive working relationships and fostered critical thinking.

**Contribution:**

This article contributes to a better understanding of ways in which digital storytelling can be used in knowledge-sharing strategies to promote disability inclusion.

## Introduction

Community-based researchers look for ways to share their research with people in other spaces such as students, clients, patients, organisations and key decision makers. The production of short, well-planned digital stories is one way to convey research information to a wide range of audiences.

The Partnerships for Inclusive Research and Learning (PIRL) was a 4-year international project focussed on participatory research and the digital divide in inclusive research. The digital divide refers to the ways in which some groups do not have access to digital systems and resources in the ways that others do (Arakpogun et al. 2020; GSMA 2022; Heeks et al. 2022; Scanlon 2021). The digital divide is larger for people with disabilities, people who live in poverty, and those who live in the Global South, as limited financial and systemic infrastructure further compound the differences in access (Datoo & Kurio 2021; Fang et al. 2019; Johansen et al. 2021). The PIRL Network was made up of over 100 community-based and academic researchers, students and professionals, who were persons living with and without disabilities from several locations around the world (primarily Cameroon and Canada) and committed to a participatory community of practice (CoP). The PIRL initiative had a focus on research that included people living with disabilities as researchers and particularly explored the roles of Information and Communication Technologies (ICT) in the social learning of researchers and research teams.

Partnerships for Inclusive Research and Learning members embarked on innovative knowledge translation (KT) and knowledge mobilisation (KMb) strategies to get key messages to a wide range of people. Digital storytelling (DST) was one of the knowledge-sharing approaches chosen by the PIRL team. A key contribution of the PIRL project to the field of digital storytelling as a KT and KMb strategy stems from the inherent diversity within its team. The project aims to reflect the varied perspectives and voices of its members, offering a richer, multi-faceted view of the issues addressed.

## Knowledge translation and knowledge mobilisation

Over the past two decades, there has been increased attention related to how knowledge and evidence, including research findings, can be used to influence policy and practices in African settings (Oronje et al. 2022; Serge et al. 2023; Uneke et al. 2022). Knowledge translation and KMb are activities that lead to the production and use of research results in ways that make them more usable and understandable to other researchers, decision makers and community members. In this article, we use the terms KT and KMb to refer to the processes for implementing research and evidence-based knowledge by researchers and community members to improve real-world conditions (SSHRC 2021). For much of our work, the goals of KT and KM have to do with social change and the development of more just and equitable social conditions. Knowledge translation and KMb both involve multiple stakeholders, including researchers, practitioners, policymakers and community members in the processes of disseminating the knowledge and evaluating its uptake and impacts (Gervais et al. 2023; Graham 2006). Both KT and KMb are usually part of academia, for example, by informing or improving research agendas, theory or methods; in other parts of society, KT and KMb contribute to public discussions and debates, policy development or service improvement (SSHRC 2021).

We make a distinction between KT and KMb in terms of scope and focus, methodology and setting. The term KT is more commonly used in the health and medical sciences, and includes the systematic review, synthesis, dissemination and application of knowledge, particularly in health-related contexts (Curran 2011) and tends to use a more linear approach from research to practice. For example, KT can emphasise translating research findings into formats that can change clinical practices and policies. Typically, KT involves more structured and formalised processes, such as creating clinical guidelines or policy briefs for a specific sector or population. Knowledge translation may include specific strategies for different stages of research translation (e.g. synthesis, dissemination and implementation) (Asamani & Nabyonga-Orem 2020).

Knowledge mobilisation is often seen as a broader term that is used in many fields including health sciences, education, social sciences and community development (Gervais 2023; SSHRC 2021). Knowledge mobilisation focusses on creating connections between the researchers (the knowledge producers) and potential users, to facilitate the use and exchange of knowledge in less linear ways. Knowledge mobilisation can include a wider array of activities and collaborative projects such as the creation of clear language documents, the development and implementation of workshops and events that prioritise community engagement, and social media campaigns. Knowledge mobilisation emphasises the co-creation of knowledge and ongoing interactions between several different groups, and can sometimes be seen as a longer-term approach. Knowledge mobilisation generally reflects the perspective that knowledge is exchanged within societies in different ways, resulting in more holistic approaches to integrating knowledge into practice than KT. Both KT and KMb include summarising and synthesising information in ways that are easier to read or view and sharing information in a wide range of both established and innovative ways.

End users such as community members, patients, families and others can be involved in the creation of KT and KMb products such as stories, videos, dramas, short documents and policy briefs. While the involvement of end-users in KT is crucial for enhancing research relevance and applicability, significant challenges hinder effective participation. The literature identifies barriers such as limited understanding of KT and KMb processes, demanding engagement requirements that strain both end-users and researchers and institutional obstacles such as bureaucratic red tape and inadequate organisational support. Documented across various contexts, these challenges suggest that the demands of involvement may outweigh perceived benefits, resulting in diminished or superficial engagement (Asamani & Nabyonga-Orem 2020; Harvey et al. 2015; Kalbarczyk et al. 2021; Lavis et al. 2006). Specifically, in low- and middle-income countries (LMICs), these barriers are exacerbated by additional constraints such as inadequate access to digital tools and Internet connectivity (Kalbarczyk et al. 2021).

In African countries, various KT structures facilitate integrating research into policy and practice. These range from institutional systems managed by national research institutes to annual events and conferences disseminating evidence. Countries such as Rwanda have integrated KT into routine health sector reviews, while Senegal has launched capacity-building programmes for policymakers and researchers. However, challenges such as inadequate funding, poor synthesis of research findings and insufficient personnel hinder these platforms’ effectiveness and impact (Asamani & Nabyonga-Orem 2020). Addressing these challenges and exploring alternatives like storytelling is essential to enhance the effectiveness of KT and KMb initiatives.

## Indigenous knowledge production in Africa

Indigenous knowledge production is a localised epistemic endeavour developed within specific communities to understand and address life’s challenges. It encompasses a wide range of accumulated strategies, practices, techniques, tools, beliefs and values unique to a particular area and influenced by the community’s worldview, socio-economic conditions and ecological context (Emeagwali 2014; Kaya 2014; Mapara 2010). This knowledge reflects the unique cognitive approaches of people who have historically inhabited an area before the introduction of dominant external cultures.

Before the colonial era, indigenous knowledge in Africa, transmitted through families and generations, was highly valued (Abdulrahman et al. 2021). Oral tradition played a crucial role in passing down epistemic values, with proverbs being a significant component of this heritage, often containing philosophical insights and practical advice (Fabidun & Olatunji 2021). African knowledge was conveyed and learned through lived experiences, storytelling, parables, riddles, myths, community ceremonies, rituals, dance, music and other narrative forms or education-centred activities (Fabidun & Olatunji 2021; Ikuenobe 2018; Ngara 2007; Shokane et al. 2018), reflecting a rich complexity often overlooked by those who prioritise written traditions. Indigenous knowledge has been mistakenly perceived as simplistic and not suitable for scientific investigation because of its oral nature and its focus on people rather than easily measurable data (Masolo 2003).

Despite historical attempts by colonial forces to suppress and devalue indigenous knowledge systems, they have persisted and remain integral to the communities that develop them. These systems are dynamic, not merely a collection of static truths but evolving insights that integrate new adaptations while respecting inherited philosophical understandings (Jimoh 2018). Individuals within these communities contribute to and reassess communal knowledge, ensuring its relevance and applicability to changing circumstances.

In recent years, there has been a growing call for the decolonisation of knowledge production (Heleta 2016; Mbebe 2016; Smith 1999) emphasising the importance of recognising and valuing indigenous knowledge systems. However, practical approaches to decolonising knowledge have received less attention. Digital Storytelling presents a valuable opportunity to achieve this, by providing a platform for the preservation and dissemination of indigenous knowledge.

The evolution of DST over the past decades in various social media spaces has provided opportunities for African storytelling to thrive. The use of DST across the continent is now widespread, with Griots, Jeli, journalists and activists, including people with disabilities, narrating their diverse stories using digital tools. By combining recorded audio narration with graphics, text, videos, pictures and music, modern-day storytellers can quickly share their stories with a wider audience and have millions of views.

## Disability-inclusive knowledge translation and knowledge mobilisation in Africa

The need for KT and KMb related to African disability stories also needs to be emphasised. Digital storytelling is a powerful tool for KT and KMb related to disability and social inclusion. Primarily, there are no ‘voiceless’ people, despite statements that are often made about ‘giving voice to the voiceless’. People with disabilities are not voiceless; rather, their voices have been silenced and oppressed by lack of opportunities and inclusive stages and spaces. For instance, there are so many untold beautiful, inspiring and empowering stories of people with disabilities in Africa that people have not listened to. This lack of interest can be partly attributed to the significant gaps in access to technology and knowledge of how to use digital tools for the majority of people with disabilities in the continent (Abrahams et al. 2023; Gould et al. 2015). Despite these challenges, many people with disabilities are now considering social media, video creation and many forms of DST as channels to promote inclusion and empowerment.

Through DST, the intersectional barriers preventing people with disabilities from participating can be challenged and broken. Particularly for women with disabilities, DST has the potential to improve access to quality services such as health, education, employment and social services. Storytelling can unlock women’s potential and contribute to a more inclusive society where every person with a disability enjoys equal rights and can thrive.

There are a growing number of people with disabilities across the African continent who are breaking the silence about disability and exclusion, and taking full advantage of digital tools to tell their stories and change the narrative. This storytelling has brought light to individual stories and collective issues affecting rights and well-being and has begun to change the way people perceive disability. Examples are captured in the African Disability Rights Yearbook which has been published for more than a decade (Ngwena 2023).

## Knowledge translation and knowledge mobilisation in the Partnerships for Inclusive Research and Learning project

The PIRL project employed several types of KT and KMb strategies throughout the 4 years of the project. The team emphasised webinars, a website, blogs and discussions to share information. In the third and fourth years, the team decided to expand its reach by focussing on various additional types of KT and sharing, including academic papers, podcasts, educational activities that used theatre and performances, the development of a Verbatim Theatre workshop about inclusion on research teams, visual arts such as a mural, the development of a newsletter, and the use of DST.

## Digital storytelling

Digital storytelling is a specific form of storytelling that allows the sharing of personal narratives in a captivating format prepared by participants in a project (not an external professional) (Lambert 2012; Park, Forhan & Jones 2021). This DST format includes a story used to share knowledge about a phenomenon, several still images with voice-over narration and background sound, and each story is two to five minute duration. Creation by project participants is crucial as it enables them to share facets of their everyday life stories, reflecting the foundational goal of including participants in key aspects of knowledge generation and sharing (De Jager et al. 2017). The storyteller has control over the way the story is told by choosing the words and images, which makes the process of production and learning meaningful for the storyteller.

There are several components of a digital story which should be included. These components are: (1) A scripted layer of about 250–375 words. The script will be read in audio and should result in a narration of about three to four minutes duration. (2) The audio layer is the recorded voice-over of the person reading the script, which becomes integrated with sounds, music or ambient noise. (3) The visual layer is made up of about 20 images with the words from the script added as captions. The person doing the final editing has to ensure that all the components fit together and that the pacing and rhythm of the text, pictures, captions and sounds or music are all aligned (De Jager et al. 2017).

## Purpose

The purpose of this article is to illustrate how DST can be done in a group setting and can be utilised to share the results of research studies, particularly for contributing to social change. A secondary purpose is to emphasise that developing digital stories with a diverse range of community members, including members of disability, academic and arts communities, can strengthen the impact of the stories.

## Research methods and design

When the PIRL team decided to integrate DST into KT and KMb processes, an invitation was extended to all network members to participate in crafting digital stories. Seven individuals agreed to participate in this initiative. Demographically, the group included six Africans from Cameroon and Sierra Leone, one white Canadian, two individuals identifying as persons with disabilities, four women, and three men. Two members were based in Canada for the duration of the project, while the remaining five resided in either Cameroon or Sierra Leone.

The project was led by a coordinator who also participated as a member, playing a crucial role in disseminating resources, leading workshops and managing logistics such as meeting schedules and deadlines. Embodying the participatory ethos of DST, the process was highly collaborative. Decisions – from scheduling to platform selection – were made democratically based on input from the coordinator and team members. The group utilised existing resources to develop our guidelines, which were then applied in our collective work. Content decisions for digital stories, such as image and sound selection, were made collectively, while individual storytellers had autonomy within established guidelines.

### Phase 1: Meeting to learn about digital storytelling

The first phase was taking time for the group to get to know each other and to learn about the specifics of how the digital stories would be developed. During this phase, we discussed the logistics, such as the technical needs, what kinds of video editing would be required and how pictures could be selected and compiled. We watched the digital stories that had been done by the Social Justice on Youth Voices group in South Africa (Walker & Martinez-Vargas 2020).

We decided to use a Google Drive folder for scripts, images and audio so that all members of the group could easily access others’ work to provide feedback. Group members also needed to talk through the use of laptops and phones, Internet access and how the voice recordings would be done. We learned who in the group had video editing tools and skills.

We decided that the timeline would include a few weeks to draft scripts and compile pictures (copyright-free, permission granted or photos that had been taken personally). Although it would come later, it was also important for group members to begin to think about the soundtrack and audio voiceover they wanted for their story. The group decided to have weekly asynchronous check-ins to share progress through a WhatsApp group, with synchronous meetings as needed.

### Phase 2: Brainstorming ideas through a Story Circle

Each participant was able to talk about ideas for their story through a Story Circle and brainstorming session. Story Circles are group activities that help participants develop, clarify or structure the digital story they want to create in a supportive group environment. In our project, the story circles were done online, and the importance of maintaining confidentiality was discussed among the team members. Each person shared personal stories within the group, which provided opportunities to explore a range of ideas, learn from each other and polish each piece.

During our first Story Circle activity, each person initially had approximately eight minutes to share their ideas and get feedback. Each person presented their rough story idea, including a draft of the script or idea sequence, and could include suggestions for visuals to go with the story. They then received feedback from the other members of the group. Group members were asked to consider the following questions when giving feedback:

- How does the story relate to the PIRL goals, to Disability Inclusive Development, to Global South-North research and ideas of equity and social justice?
- Who is the intended audience for this story?
- What first-hand experience could be included to convey their ideas to that audience?
- What aspects of the story could be expanded upon?
- What could be clearer?

During this phase, there was an emphasis on supportive discussion, and members all agreed that hearing the stories that their colleagues felt were important was a very meaningful process.

### Phase 3: Drafting the scripts

Group members worked individually or in pairs to develop their scripts. When they were ready with a draft, they informed the other group members, who then reviewed and provided feedback. This iterative process continued until each of the final scripts was finished.

### Phase 4: Finding, compiling and matching pictures to script and identifying background music

When the script was done, the next step was to select pictures and music to accompany it. We set ground rules about what could be used (e.g. they had to be copyright-free). We developed a library where pictures were shared and saved, and each person also sourced their own pictures from various places. When someone could not find a desired picture or was not clear on what image to use for a particular segment, they would ask the group for assistance. Music was also found using several different avenues.

### Phase 5: Recording and editing the final digital story

Once the written scripts were finalised and the pictures had been decided on, the audio recording was made. Edits were made to ensure that the timing was correct, the text of the script was added as captions, and that the audio and text corresponded to the pictures. This step took editing skills and time to ensure that the final digital story was polished and within the parameters that had been predetermined (i.e. within about three minutes, the content fit with the project goals, pictures corresponded to the script, captions matched the audio). There was considerable discussion about details at this phase, which required ongoing encouragement so that the final videos would be completed.

### Phase 6: Sharing final outputs and reflections on the process

In the final phase, we ensured the digital stories were uploaded to our YouTube channel for sharing. We then presented these stories to other team members and our wider network. Reflecting on the process assisted us in appreciating the work and what could be done similarly and differently in similar future projects.

### Ethical considerations

Ethical clearance to conduct this study was obtained from the University of Bamenda, Ethical Review Committee (No. 2019/0124H/Uba/IRB) and the University of Toronto, Health Sciences Research Ethics Board (No. 39308). The digital stories were being developed by members of the research team (not by other community members), no additional ethical approvals were required for this project.

## Results

The group created five digital stories (Table 1) in video format (with captions) which are publicly available online. Each one addresses a different aspect related to disability inclusive research, with many having a focus on Africa and the creation of African evidence. The importance of assisting community members to think about and support research and evidence creation was one of the goals of the project, and the videos reflect this goal. The stories are available at this link: https://www.youtube.com/playlist?list=PLVz3uqJ8UbEvLpQ54LWnS21swm2kK1rL0.

**TABLE 1:** Overview of the digital stories created in the Partnerships for Inclusive Research and Learning project.

Story title	Description
The PIRL Story	Different voices narrate some perspectives on the complexities of international disability-inclusive research teams.
Coordinating an inclusive research team	The perspective of an African research project coordinator, and the challenges he faced in ensuring the PIRL project went well.
Roles of associations of persons with disabilities in research	This story describes the leadership and the possible influences of community-based organisations, particularly organisations of persons with disabilities, in research teams from the perspective of a group member who has seen successes and difficulties.
Grieving from within	Two women leaders talk about how they have come to see the importance of research when engaged in advocacy for women’s rights and combat gender-based violence in the disability community.
My journey into therapeutic theatre	A student talks about how his question of ‘But what can I do?’ led to understanding and using different aspects of therapeutic theatre, including the importance of evaluation and research.

PIRL, Partnerships for Inclusive Research and Learning.

The videos provide an avenue to share insights about disability-inclusive development research. Group members stated that the beginning part of the process of creating the digital stories significantly improved their understanding of what to consider when translating evidence into formats that are more understandable.

The five stories explored themes of disability-inclusive research and why research is important in community building and development; aspirations for better research support and evidence creation in Africa; partnerships across local and international teams; and social justice as foundational to disability-inclusive research.

## Discussion

In alignment with previous research (Cunsolo Willox, Harper & Edge 2013; Lindvig 2017; Manning 2010), DST has shown to be an appropriate methodology for a participatory research project such as the PIRL project. As a research method, DST holds promise as a decolonising tool because it does not focus on representations of marginalised groups from the dominant perspective. Digital storytelling empowers storytellers by placing the power of expression and knowledge creation directly in their hands, thereby supporting the decolonisation of narratives and the knowledge they embody. This method empowers participants to convey their stories authentically, utilising their own voices, perspectives and chosen representations (White & Epston 1990). Furthermore, DST can tap into a rich and diverse tradition of storytelling that has historically played a crucial role in mobilising change within communities and networks (e.g. Abbenyi-Nfa 2007; African Literature Association 2013). By doing so, through not only the creation of knowledge but also in its delivery, DST has been proven effective in ensuring that marginalised perspectives are preserved and continue to thrive in various contexts (Perley 2009; Rieger et al. 2021).

In practice, the PIRL project’s diverse group, spanning different countries, genders, abilities and backgrounds, enriches our DST. This diversity shaped the DST process, ensuring that narratives were constructed ‘from the inside out’, capturing the authentic voices and perspectives of its participants. For instance, in creating ‘The PIRL Story’, integrating these varied voices was paramount to depict the complexity and richness of the experiences shared. This approach not only highlighted the individual contributions but also how they collectively formed a nuanced and comprehensive portrayal of the group’s dynamics and insights. This methodology highlights DST’s role as a powerful platform for inclusive representation. By utilising the diverse backgrounds of its members, the PIRL project demonstrates how DST can foster inclusive representations of community and scholarly experiences. This approach not only emphasises DST’s use for KT and mobilisation but also amplifies the diverse voices within the project, enhancing the discourse and understanding of the issues addressed.

While the outcome of DST is undoubtedly significant, existing research shows that the process of creating it can sometimes be equally, if not more, important (Brushwood Rose & Granger 2013; Gubrium et al. 2016; Rich, Lamola & Woods 2006; Wexler et al. 2013). In the context of our DST, the story circles emerged as a particularly valuable aspect of the process that warrants further discussion. At the outset of creating our stories, we organised a story circle. Although later sessions were not explicitly labelled as story circles, they used the same format: team members shared their stories, intentions and messages, and received feedback from others. These sessions offered us opportunities to reflect on our journey within the PIRL project and other research-related experiences. They enabled a holistic examination of our experiences, considering the beginning, end, the various highs and lows, as well as the lessons learned along the way.

The process of using narratives to share experiences, as in the case of digital stories, can be emotionally demanding work (Boydell, Solimine & Jackson 2016). However, several benefits were observed, aligning with prior studies that highlight the advantages of DST (Brushwood Rose & Granger 2013; Gachago et al. 2013; Ribeiro 2016; Wexler et al. 2013). In our project, story circles not only provided opportunities to scrutinise and make sense of our experiences but also facilitated meaning-making. The creation of these digital stories promoted deep reflection on our personal experiences and the experiences of others, fostering empathy and understanding of complex situations. The process encouraged critical and creative thinking and led to group members having very personal and vulnerable conversations. Through engaging in this process, we realised the critical roles that creating and sharing digital stories can play in sharing knowledge, advocating for change and amplifying voices from African contexts. One of the group members said:

‘I based my story on my experiences and found it helpful to have others to assist me to not be too emotional in my writing. I learned a lot, and was surprised at how the stories developed as the pictures were added. Being involved in the process made me think about how important storytelling can be. I also learned about how projects can be coordinated and managed. The project was organised so that there were challenges but not too much difficulty, and we understood what had to be done step by step. We were encouraged to ask questions when we were not clear on what needed to be done, and have discussions with the others. Being involved in a community-based, inclusive research project showed us that it can be done and can make a difference.’ (Cameroonian male, academic, community organizer)

### Connecting to other digital storytelling in Africa and other places

Our DST project primarily builds upon the work of Melanie Walker, Carmen Valquez Garcia and the graduate students they collaborated with in South Africa on the ‘Youth Voices on Social Justice’ project (Walker & Martinez-Vargas 2020). As we developed our own digital stories, we found ourselves continually returning to both their website and the session they had done with us as part of the PIRL Institute 2021, as their directions and enthusiasm were very helpful.

Others have also used DST in Africa or related to disability, and reviewing their work reveals similarities and differences in what we did. Variations are especially evident in the process; although there is a framework, there is not just one way of using it. One collection of digital stories related to disability inclusion and human rights was produced by the Secretariat of the African Decade of Persons with Disabilities (SADPD 2011). These stories use a similar process to the PIRL stories, to reflect real-life efforts of people with many different experiences, and from many African countries; by telling their stories, the creators aim to inspire others and to improve the attainment of human rights.

Some of the challenges we experienced with technology were identified by Yan and colleagues (Yan et al. 2021), who also cited Dreyer et al. (2017) and Gogela and Ntwasa (2015) in talking about the challenges of navigating video editing software in African contexts. The selection of video editing tools and processes appears to be a particularly relevant consideration in African contexts, where training, Internet access and electricity outages can compound other technical challenges. Another example of DST related to disability is provided by Sitter and colleagues who report on a project titled ‘My life. My story’, which focussed on transitions for adults with developmental disabilities (Sitter et al. 2023). Nonetheless, as our project and the others cited here indicate, access to and expertise in the use of technology continues to improve in many places, making DST more accessible and feasible, and an engaging KT and KMb tool in African contexts.

### Reflections

Embarking on the DST project was a successful part of the overall project, particularly in disseminating information about project activities. This type of project involves numerous details, making the role of a project coordinator essential for maintaining organisation and progress. Creating the digital stories proved more challenging than some participants had anticipated. The process was time-consuming and occasionally led to frustration among team members, highlighting the significant commitment required to complete the stories. The project coordinator’s assistance in fostering teamwork and maintaining motivation was crucial. Additionally, having a team committed to the project’s goals and activities, supported by the coordinator, was instrumental in ensuring the completion of the digital stories.

From the DST project, group members learned new digital skills in storytelling, including how to do voiceovers, and how to write, review and edit a script. Leadership skills were also improved. As one member said:

‘As a woman with a disability, this process helped me learn about how to coach other women with disabilities to take part in storytelling. I understood the role of a director in doing a short film.’ (Cameroonian female, community leader, living with a physical disability)

And several members talked about being more comfortable with taking leadership in projects. Some members have continued to use DST, both using the methods described here and other approaches, in their work. For example, one member has written and edited over 10 digital stories for local organisations which have reached other collaborators, partners and funders. Some of these were to report the activities and findings from various projects, and others were for advocacy. Some members have gone on to use digital stories in other ways, including to promote their work, gain social media visibility, and improve access and opportunities for others in need. Other local organisations have shown interest in DST because they have seen the evidence of their impact.

The process as a whole was part of the broader PIRL initiative, which aimed, among other objectives, to include women and girls with disabilities in research and community-based learning. The DST project is one example of how women can benefit from technical and financial support to improve their inclusion and participation in inclusive research and learning processes in Africa. We realised that it was a way to reach beyond local women with disabilities to others in Africa and globally. It provided an example of how women can become research assistants and researchers themselves when they are provided the support they need. They have relevant skills to be used in research and often have better mastery of local languages used in their communities than the researchers who come from outside. Future work could develop these skills and aspirations, including developing digital stories in other languages.

One strength of DST, which is not unique to the African context, is its ability to easily reach a wide audience, both locally and globally, thus contributing to the growth of a rich body of knowledge. By harnessing the power of digital tools, digital storytellers can bridge geographical distances, and connect with people from different backgrounds. The relative simplicity of digital stories allows for better access by many in communities with less reliable electricity and Internet access, as compared to other forms of digital content such as full-length documentaries and films which may require longer times to create, more financial resources, better connectivity and storage capacity on smartphones. They mitigate against the limitations posed by other forms of knowledge dissemination such as papers and books, as those who might not be able or willing to read can watch and understand digital stories.

### Limitations

A significant limitation of this article was the lack of systematic data collection although the project was part of a larger participatory research project. The data presented are primarily retrospective, as the DST activity was not originally conducted with the intention of producing a research article. This retrospective approach led to several constraints: (1) Incomplete data: some of the data about what happened may be missing or incomplete because it was not collected with research objectives in mind. (2) Inaccuracies and recall bias: gathering data retrospectively can introduce inaccuracies and recall bias, and in our situation this is possible. Especially given how time-consuming the DST process is, we might have missed some key aspects in recounting what was done.(3) Methodological limitations: although we did keep written records of our process, we did not collect personal notes from the group members. The informal nature of data collection regarding each person’s perspectives later in the project meant that rigorous methodological approaches were not consistently applied. However, maintaining extensive records of meetings, discussions and reflection sessions was beneficial in writing this account. We recommend that future teams, if resources permit, conduct DST in a manner that facilitates the systematic documentation of both the process and the results, ensuring a more robust foundation for subsequent research.

The DST process, while a powerful medium for narrative expression, introduces several challenges that can impact the authenticity and integrity of the stories produced. Conducting story circles, a common technique in this process, presents unique difficulties. While participants are expected to own their stories, speak in their voices and share what they want, story circles require them to present their ideas to others, discuss them and receive feedback. This dynamic ultimately resulted in multiple participants influencing the content of the stories, which was witnessed in both the individual and group stories in our project. In the group stories, multiple people contributed to every content decision, including what to discuss, the final script, the choice of photographs and the music. For the individual stories, while the main author primarily decided on the topic, other aspects of the DST process were influenced by group members.

Determining whether this influence is an advantage or disadvantage remains unclear, as the degree of group influence on an individual’s story was never assessed. This raises questions about the limitations of collaboration in DST. Furthermore, the duration constraints of digital stories imply that while they can be used to highlight certain aspects of a story, other essential elements might be omitted. This challenge has been noted by other researchers in their experiences with DST (Boydell et al. 2016; Brushwood Rose & Granger 2013). Overall, while story circles provide valuable opportunities for reflection and feedback, they can also complicate the storytelling process by introducing additional layers of consideration and influence.

Digital storytelling also introduces significant barriers, particularly because of the digital divide, which can disproportionately impact certain groups, including persons with disabilities and individuals in lower economic contexts. Digital storytelling requires specific technological tools such as computers, software for video editing and reliable Internet access, which are not universally available. Many of the group members were working in locations with limited or sporadic electricity and Internet access. There were several challenges for people in Cameroon and Sierra Leone to have adequate Internet access to easily search for pictures, music and audio, and to do editing, particularly in Cameroon where electricity and Internet were often interrupted, sometimes for long periods of time. At times, the extensive background work required for DST seemed disproportionate to the benefits it yielded, suggesting that this method might not be suitable for all projects. We found that we often had to go between more than one mobile device and use power banks to be able to deal with power issues. To mitigate the challenges with Internet connectivity, switching between several network providers depending on which one was more reliable at any given point in time was also necessary. Often extra patience by all team members was needed to wait for certain times to upload or download data and files.

Nevertheless, these barriers are not unique to our situation but are indicative of a broader systemic issue. Many communities lack the necessary infrastructure for high-speed Internet and the educational frameworks to foster digital literacy. Digital storytelling requires more than just narrative skills; it demands proficiency in using digital tools for editing and production. These challenges are further compounded for persons with disabilities, as accessibility issues can severely limit their engagement with digital platforms. As a result, while DST is a powerful tool for advocacy and self-expression, it can unintentionally reinforce social and economic disparities by favouring those with existing technological access and skills (Lythreatis, Singh & El-Kassar 2022).

Having grappled with the challenges of making digital stories more accessible, particularly for African audiences, we believe that they can be a useful tool in KT and KMb activities. One important aspect is to ensure that digital stories are inclusive of people with various disabilities, allowing them to be accessed and enjoyed by all. The transcripts can be used by people with vision impairments and hearing impairments to access the storyline. However, we did not do detailed alternative text descriptions of each picture as we did not have the time to do that, or the financial resources to pay someone to do it. This lack of description meant that those who could not view the stories missed some of the impact. With each digital story having many pictures, it would have been over 100 pictures that needed to be described. One recommendation would be to build in a process of adding the written description of pictures as they are chosen so that it might not take as long, or to include a budget item for someone to have dedicated time to provide descriptions.

All of our digital stories are available on YouTube, which allows us to track the number of views. We have been disappointed that to date there has not been more interest in them, as the viewing numbers are small. We do know that some of the stories were shared in other ways such as on WhatsApp and in in-person meetings, and so these numbers do not reflect the total number of views. Our project did not have large amounts of funding to promote the digital stories and to encourage educators and community workers to use them. If resources were available, it would have been beneficial to have some strategies to ensure that the stories were well-publicised. We encourage those who are applying for funding to do DST to include dissemination in their project proposal and budget.

## Conclusion

This article underscores the potential of DST as a potent participatory research method and a mechanism for decolonising knowledge production. It highlights how DST empowers marginalised groups by enabling them to articulate their narratives from their own perspectives, thereby challenging dominant narratives. Additionally, it illustrates how the diversity within research teams, exemplified by the PIRL project, can enhance the storytelling process, resulting in more authentic and inclusive representations of experiences. The article also addresses the practical challenges of DST, particularly in African contexts, where technological limitations and accessibility issues may impede its application, thus highlighting the need for innovative solutions to bridge the digital divide and ensure that DST remains an inclusive and effective tool for KT and mobilisation.

Overall, both the DST group and the entire PIRL team expressed high satisfaction with the digital storytelling process and its outcomes. The creation of digital stories requires significant commitment, time, access to digital tools and financial resources. Our team found the collaborative process to be meaningful, resulting in a long-term, tangible product that effectively communicates aspects of this complex project to a broader audience. Creating the digital stories encouraged positive working relationships and critical thinking. The final videos are a tangible and useful way to share information with others.
